# Risk prediction and clinical utility analysis of postoperative pancreatic fistula: a comparative study of multivariable logistic regression and random forest models

**DOI:** 10.3389/fsurg.2025.1596224

**Published:** 2025-06-13

**Authors:** Kaixuan Zhang, Kunlun Chen

**Affiliations:** Department of Hepatobiliary Surgery, The First Affiliated Hospital of Zhengzhou University, Zhengzhou University, Zhengzhou, China

**Keywords:** postoperative pancreatic fistula, pancreatoduodenectomy, surgical complications, random forest, machine learning

## Abstract

**Objective:**

Compare the performance of the Multivariable logistic regression (LR) model based on traditional statistical methods and the Random Forest (RF) model in machine learning for predicting clinically relevant postoperative pancreatic fistula (CR-POPF) after pancreatoduodenectomy (PD).

**Background:**

CR-POPF is a common and severe complication following PD. Traditional statistical models are widely used to predict it, but the rise of machine learning has garnered attention for its potential in predictive medicine. Comparing the performance of traditional statistical methods and machine learning models provides insight into the optimal approach for CR-POPF prediction.

**Methods:**

Clinical data from patients undergoing PD were collected. CR-POPF prediction models were developed using Multivariable LR and RF, and their predictive performance was compared using Calibration curves, ROC curves and DCA curves.

**Results:**

In the calibration curve analysis, the Multivariable LR model shows better calibration than the RF. The Multivariable LR model achieved an AUC of 0.96, while the RF model achieved an AUC of 0.90, indicating superior predictive accuracy of the Multivariable LR model. Decision curve analysis demonstrated that the Multivariable LR model provided higher net benefit across most threshold ranges than the RF model.

**Conclusion:**

The Multivariable LR model outperformed the RF model in predicting CR-POPF after PD and can be considered the preferred method for CR-POPF risk assessment.

## Introduction

PD is a commonly used surgical approach for treating benign and malignant diseases of the pancreatic head, distal common bile duct, and periampullary region (1, 2). One of its most dreaded complications is CR-POPF, which occurs in 10%–20% of patients and is associated with higher mortality, delayed gastric emptying, infections and bleeding, prolonged hospital stays, increased costs, and unplanned readmissions (3–5). How to accurately predict CR-POPF remains a pressing challenge for clinicians. Precise prediction of CR-POPF facilitates risk stratificationand the development of personalized treatment strategies for patients undergoing PD.

Machine-learning methods, especially ensemble algorithms such as the Random Forest, have recently been introduced into high throughput omics data because they model complex nonlinear interactions that conventional LR may overlook (6–11). However, its performance and relevance studies with conventional clinical data have not been thoroughly evaluated. For CR-POPF, no study has quantified whether RF outperforms the widely used multivariable LR scores for CR-POPF (11, 12). The original Fistula Risk Score and its alternative version, and several single-centre nomograms built with multivariable LR are widely used to predict CR-POPF; however, their discrimination (AUC = 0.70–0.85) declines in heterogeneous cohorts (13, 14). In other studies that developed CR-POPF prediction models using logistic regression, fewer variables were incorporated, and the assessments were rather superficial, so the clinical benefit remains uncertain (15, 16). Here, we construct both models in a 289-patient East-Asian cohort and compare their discrimination, calibration and decision-curve performance to determine the more clinically useful strategy for CR-POPF risk stratification.

## Methods

This study was conducted in accordance with the Declaration of Helsinki and approved by the Ethics Committee of the First Affiliated Hospital of Zhengzhou University (Approval No.: 2024-KY-0532-001), with a waiver of informed consent. A retrospective analysis was conducted on the clinical data of patients who underwent PD surgery across all campuses of the First Affiliated Hospital of Zhengzhou University from January 2019 to March 2024, with routine postoperative follow-up conducted. The surgical approaches included OPD (Open Pancreaticoduodenectomy), LPD (Laparoscopic Pancreaticoduodenectomy), and RPD (Robotic Pancreaticoduodenectomy).

Inclusion criteria: (1) Patients undergoing PD treatment for benign or malignant tumors around the ampulla. (2) Generally in good physical health, specifically defined as: Left ventricular ejection fraction ≥50%, no recent myocardial infarction or unstable angina within 6 months; No recent stroke or transient ischemic attack within 6 months, no uncontrolled epilepsy or cognitive impairment; No severe chronic obstructive pulmonary disease, GOLD stage III–IV, Forced Expiratory Volume in 1 s [FEV1] ≥60% predicted; estimated glomerular filtration rate ≥60 ml/min/1.73 m^2^, no requirement for dialysis. (3) No prior systemic anti-tumor treatments before surgery, including but not limited to chemotherapy, radiotherapy, or immunotherapy. (4) Complete clinical and follow-up data are available.

Exclusion criteria: (1) Vascular involvement. (2) Distant metastasis of the tumor. (3) Special intraoperative situations: Patients converted from laparoscopic or robotic surgery to open surgery. Patients undergoing combined resection of other complex organs (e.g., spleen, major vascular reconstruction). (4) Special populations: Pregnant or lactating women. Children (<18 years) or elderly patients (>80 years).

Included patients: After applying the inclusion and exclusion criteria and considering dataset balance, 289 patients were included. 137 patients were in the CR-POPF group, and 152 patients were in the non-CR-POPF group.

The clinical variables included in our prediction model were selected based on previous literature and clinical experience (17, 18). Observation indicators: (1) Preoperative demographic characteristics of patients: age, gender, body mass index (BMI), smoking history, drinking history, preoperative jaundice status, history of heart disease, hypertension, diabetes, upper abdominal surgery, and ECOG score. (2) Preoperative laboratory test-related variables: preoperative blood sample levels (i.e., hemoglobin level, white blood cell count, neutrophil/lymphocyte ratio), plasma total bilirubin level, and related tumor markers, namely carbohydrate antigen 19-9 (CA19-9), cancer antigen 125 (CA125), and carcinoembryonic antigen (CEA). (3) Perioperative-related data: preoperative bile drainage, ASA score (American Society of Anesthesiologists), total operative time, surgical approach, estimated blood loss, intraoperative plasma transfusion volume, intraoperative red blood cell transfusion volume, and number of lymph nodes dissected. (4) Intraoperative evaluation of tumor location, pancreatic texture, and pancreatic duct diameter.

Selection of included variables: This study focused on preoperative and intraoperative factors affecting the occurrence of pancreatic fistula to construct a risk prediction model applicable during or before surgery, aiming to guide early clinical interventions. Therefore, only preoperative variables (e.g., age, gender, BMI) and intraoperative variables (e.g., pancreatic duct diameter, pancreatic texture, intraoperative blood loss, surgical duration) were included in the analysis. Postoperative variables (e.g., duration of intravenous analgesic use, amylase levels in drainage fluid) were not included in this study, as they are only available postoperatively, making them unsuitable for preoperative risk assessment and potentially a result rather than a cause of pancreatic fistula.

The classification of pancreatic fistula is primarily based on the criteria proposed by the International Study Group on Pancreatic Fistula (ISGPS) (19). Based on the clinical impact and required interventions, postoperative pancreatic fistula is classified into three grades: Biochemical Leak: No clinical significance; observation only is required. Grade B fistula: Requires additional treatment but poses no life-threatening risk. Grade C fistula: Severe; requires urgent intervention or surgery and is life-threatening. Clinically relevant POPF (CR-POPF) includes Grade B and Grade C fistulas, while Biochemical Leak or the absence of a fistula are classified as non-clinically relevant pancreatic fistula (non-CR-POPF). The pancreatic texture was classified retrospectively from the original operative notes recorded by the attending surgeons. In these operative records, surgeons explicitly described the pancreatic texture as either soft or firm based on direct intraoperative palpation and subjective surgical judgement. No standardized instrument or quantitative measure was routinely employed for this assessment. Cases lacking clear documentation regarding pancreatic texture were excluded from analysis for consistency. The pancreatic duct diameter variable is defined as a binary variable. Positive (pancreatic duct diameter ≥3 mm) indicates a relatively wide duct, while negative (pancreatic duct diameter <3 mm) indicates a narrower duct. This threshold is based on relevant literature and clinical experience (20) and is commonly used for assessing postoperative pancreatic fistula risk.

Missing data were handled using the multiple imputation method. All imputation procedures were conducted using the MICE package in R software (version 4.3.1, R Core Team, Vienna, Austria). Descriptive statistics were performed using SPSS software (Version 22, IBM Corp., Armonk, NY, USA) to compare the baseline characteristics of the CR-POPF group (137 cases) and the non-CR-POPF group (152 cases). Normally distributed continuous variables were expressed as mean ± standard deviation (SD) and compared using the independent sample *t*-test. Non-normally distributed continuous variables were expressed as median (interquartile range, IQR), denoted as *M* (Q1, Q3), and compared using the Mann–Whitney *U* test. Categorical variables were expressed as frequencies and percentages and compared using the chi-square test or Fisher's exact test where appropriate. Univariate LR analysis was performed to identify potential risk factors for CR-POPF, with CR-POPF as the dependent variable and preoperative and intraoperative factors (e.g., age, sex, BMI, pancreatic duct diameter, and pancreatic texture) as independent variables. Results were reported as odds ratios (ORs) with corresponding 95% confidence intervals (CIs). A two-tailed *P*-value <0.05 was considered statistically significant variables demonstrating statistical significance were chosen for subsequent multivariate analysis.

Multicollinearity test: To evaluate potential multicollinearity, the Variance Inflation Factor (VIF) was employed to analyze the selected variables (21). VIF values were calculated using a linear regression model (lm function), with a VIF value <10 indicating no severe multicollinearity between variables. The analysis was conducted using R software. Primarily using the car package. Correlation Analysis (22): Correlation analysis was conducted on the selected variables. The correlation matrix was calculated using Pearson correlation coefficients, and the analysis was also conducted in R software, primarily visualized using the corrplot package.

Model Construction and Evaluation: To effectively avoid overfitting and enhance the robustness of the results, a 10-fold cross-validation approach is employed for training and evaluating the model. Specifically, the data are randomly divided into 10 folds; in each iteration, one fold is designated as the validation set, while the remaining nine folds serve as the training set. This procedure is repeated 10 times, and the average metric is reported as the final evaluation. Continuous variables were log-transformed if skewed and then standardized, categorical variables were one-hot encoded, and all preprocessing was executed within each cross-validation fold to avoid data leakage.

Multivariable LR (23) was conducted to investigate the independent effects of the selected variables on the occurrence of CR-POPF. CR-POPF occurrence was set as the dependent variable, Variables with *P*-value <0.05 in univariate analysis were used as independent variables. Results were expressed as ORs and their 95% CIs. To clearly illustrate the results of the Multivariable LR analysis, this study utilized the forestplot package in R software to generate a forest plot. The plot showed each variable's OR and 95% CIs, helping readers understand their independent roles in the occurrence of postoperative pancreatic fistula. To improve the interpretability of the forest plot, the *X*-axis was transformed using a base-10 logarithmic scale. This transformation compresses the variable range for better visualization while maintaining the original OR and CI values.

The RF (24) was constructed using 500 decision trees (ntree = 500), with 3 variables randomly selected (mtry = 3) for node splitting, to evaluate the importance of each variable. Variable importance was measured by the Mean Decrease Accuracy (MDA), reflecting the contribution of each variable to the model's predictive performance. The analysis was performed using the randomForest package in R software. To visually present feature importance, bar charts were created using the ggplot2 package, showing the ranking of variable importance.

To assess the agreement between predicted probabilities and actual event occurrence, this study employed calibration curve analysis (25) for both the Multivariable LR and RF models. Specifically, predicted probabilities were grouped into equal-sized bins, and each bin's mean predicted probability was plotted against the corresponding observed proportion of events. A diagonal line (*y* = *x*) represented perfect calibration, enabling direct visual comparison of how closely each model's predictions matched reality. The plots were generated in R using ggplot2, with larger deviations from the diagonal indicating poorer calibration performance.

To evaluate the predictive performance of the models, Receiver Operating Characteristic (ROC) curve analysis (26) was used to compare the classification accuracy of the Multivariable LR model and the RF model. The ROC curves were drawn by matching predicted probabilities with actual pancreatic fistula outcomes, and the area under the curve (AUC) was calculated using the pROC package in R software to measure the discriminatory power of the models. The ROC curves were visualized using the ggplot2 package, with the AUC displayed as annotations on the plot. Model performance was compared based on AUC values, with higher values closer to 1 indicating better predictive accuracy.

To further evaluate the clinical utility of the Multivariable LR model and the RF model in predicting CR-POPF, this study employed the Decision Curve Analysis (DCA) (27) method to calculate the Net Benefit of the two models across different threshold probabilities. DCA was performed using the dcurves package with a threshold probability range of 0–1. DCA evaluated the clinical utility of the predictive models by comparing the net benefits of different strategies, such as Treat None and Treat All.

## Results

### Baseline characteristics of patients

A total of 289 patients were included in this study, with 137 cases in the CR-POPF group and 152 cases in the non-CR-POPF group. The baseline characteristics of the two groups were compared (Table 1). Demographics and Preoperative Variables: There was no significant difference in gender distribution (*P* = 0.21), age distribution (*P* = 0.94), or surgical approach (*P* = 0.84) between the CR-POPF and non-CR-POPF groups. However, patients in the CR-POPF group had a significantly higher BMI compared to the non-CR-POPF group (*P* = 0.02). The prevalence of preoperative jaundice was significantly higher in the CR-POPF group (45.3%) compared to the non-CR-POPF group (32.9%) (*P* = 0.03). Smoking and drinking histories showed no statistical differences between the two groups (*P* > 0.05). Comorbidities, including hypertension, diabetes, and cardiovascular disease, were similarly distributed between the groups (*P* > 0.05). Laboratory and Tumor-Related Variables: Serum CA19-9 levels were significantly higher in the CR-POPF group compared to the non-CR-POPF group (*P* = 0.01), suggesting an association with an increased risk of CR-POPF. However, levels of CA125, CEA, and Plasma total bilirubin did not differ significantly between the groups (*P* > 0.05). Intraoperative Characteristics: Key intraoperative variables, including estimated blood loss and the number of lymph nodes dissected, were comparable between the two groups (*P* > 0.05). However, pancreatic texture and pancreatic duct diameter showed significant differences. Patients with a soft pancreatic texture were more likely to develop CR-POPF (*P* < 0.001). Similarly, a smaller pancreatic duct diameter (<3 mm) was strongly associated with CR-POPF (*P* < 0.001).

**Table 1:** Comparison of baseline, clinical, and intraoperative characteristics between POPF and non-POPF groups.

Variable	Non-pancreatic fistula group	Pancreatic fistula group	*P* value
Gender			0.21
Male	90 (59.2)	91 (66.4)	
Female	62 (40.8)	46 (33.6)	
Smoking history			0.06
Yes	45 (29.6)	55 (39.8)	
No	107 (70.4)	82 (60.2)	
Drinking history			0.69
Yes	34 (22.4)	28 (20.4)	
No	118 (77.6)	109 (79.6)	
Presence of jaundice			0.03
Yes	50 (32.9)	62 (45.3)	
No	102 (67.1)	75 (54.7)	
History of heart disease			0.99
Yes	20 (13.2)	18 (12.9)	
No	132 (86.8)	119 (87.1)	
History of hypertension			0.16
Yes	43 (28.3)	29 (21.5)	
No	109 (71.7)	108 (78.5)	
History of diabetes			0.60
Yes	28 (18.4)	22 (16.1)	
No	124 (81.6)	115 (83.9)	
History of upper abdominal surgery			0.29
Yes	14 (9.2)	18 (12.9)	
No	138 (90.8)	119 (87.1)	
ECOG score			0.54
0	81 (53.3)	76 (55.5)	
1	63 (41.4)	51 (37.2)	
2	7 (4.6)	10 (7.3)	
3	1 (0.7)	0 (0.0)	
ASA score			0.24
Ⅰ	5 (3.3)	9 (6.5)	
II	123 (80.9)	100 (73.1)	
III	23 (15.1)	28 (20.4)	
IV	1 (0.7)	0 (0.0)	
Biliary drainage			0.30
Yes	28 (18.4)	19 (13.8)	
No	124 (81.6)	118 (86.2)	
Surgical approach			0.84
Open surgery	58 (38.2)	54 (39.4)	
Laparoscopic surgery	56 (36.8)	46 (33.6)	
Robotic surgery	38 (25.0)	37 (27.0)	
Pancreatic texture			<0.001
Soft	43 (28.3)	115 (83.9)	
Firm	109 (71.7)	22 (16.1)	
Pancreatic duct diameter			<0.001
Small	20 (13.2)	100 (73.1)	
Large	132 (86.8)	37 (26.9)	
Tumor location			0.58
Duodenum or ampulla of Vater	53 (34.9)	46 (33.3)	
Pancreas	63 (41.4)	73 (53.3)	
Bile duct	36 (23.7)	18 (12.9)	
Age (years)	59.48 ± 11.47	54.87 ± 12.01	0.94
BMI (kg/m²)	22.90 ± 2.84	26.38 ± 1.81	0.02
Hemoglobin level (10^12^/L)	120.5 (107.2, 131.0)	120.2 (101.1, 126.0)	0.61
White blood cell count (10^9^/L)	6.1 (4.9, 8.2)	6.6 (4.8, 9.5)	0.53
Neutrophil-to-lymphocyte ratio	2.9 (2.0, 5.3)	3.2 (1.9, 5.4)	0.74
CA19-9 (U/ml)	41.8 (10.3, 156.3)	213.6 (65.7, 240)	0.01
CA125 (U/ml)	12.6 (8.1, 21.8)	15.4 (9.6, 20.5)	0.63
CEA (ng/ml)	3.1 (1.7, 4.9)	2.6 (1.6, 4.4)	0.57
Plasma total bilirubin level (μmol/L)	81.8 (22.1, 205.2)	79.9 (12.5, 197.0)	0.56
Surgery duration (h)	5.2 (4.5, 6.0)	4.9 (4.4, 5.6)	0.52
Estimated blood loss (ml)	200 (150, 400)	300 (150, 550)	0.61
Intraoperative transfusion volume (ml)	0.0 (0.0, 0.0)	0.0 (0.0, 450)	0.09
Intraoperative red blood cell transfusion volume (U)	0.0 (0.0, 0.0)	0.0 (0.0, 2.0)	0.08
Number of lymph nodes removed	9.0 (6.0, 11.0)	8.0 (5.0, 14.0)	0.84

BMI represents body mass index; ECOG represents the eastern cooperative oncology group; ASA represents the American society of anesthesiologists; CA represents carbohydrate antigen; CEA represents carcinoembryonic antigen.

### Univariate LR analysis

Univariate LR analysis was performed to evaluate the relationship between various preoperative and intraoperative factors and the occurrence of CR-POPF. The dependent variable was defined as the presence of CR-POPF, while each individual variable was analyzed as an independent variable. The results are summarized in Table 2. Higher BMI was significantly associated with an increased risk of CR-POPF (OR = 1.68, 95% CI: 1.28–2.19, *P* < 0.001). Patients with preoperative jaundice had a higher likelihood of developing CR-POPF (OR = 3.06, 95% CI: 1.03–9.07, *P* = 0.044). Soft pancreatic texture was strongly correlated with an elevated risk of CR-POPF (OR = 0.03, 95% CI: 0.01–0.08, *P* < 0.001). Smaller pancreatic duct diameter was associated with a significantly higher risk of CR-POPF (OR = 0.06, 95% CI: 0.016–0.19, *P* < 0.001). Elevated CA19-9 levels were significantly linked to an increased risk of CR-POPF (OR = 1.01, 95% CI: 1.01–1.01, *P* = 0.001).

**Table 2:** Univariate LR analysis results for CR-POPF.

Variable	*β*	S.E.	*Z*	*P*	OR (95% CI)
Gender					
Male					1.00 (reference)
Female	−0.32	0.57	−0.56	0.575	0.73 (0.24–2.23)
Smoking history					
No					1.00 (reference)
Yes	0.46	0.56	0.83	0.407	1.59 (0.53–4.72)
Drinking history					
No					1.00 (reference)
Yes	−0.14	0.67	−0.21	0.833	0.87 (0.23–3.25)
Jaundice					
No					1.00 (reference)
Yes	1.12	0.55	2.02	0.044	3.06 (1.03–9.07)
History of heart disease					
No					1.00 (reference)
Yes	0.02	0.80	0.02	0.985	1.02 (0.21–4.84)
History of hypertension					
No					1.00 (reference)
Yes	−0.94	0.78	−1.21	0.228	0.39 (0.08–1.80)
History of diabetes					
No					1.00 (reference)
Yes	−1.15	1.06	−1.09	0.276	0.32 (0.04–2.51)
History of upper abdominal surgery					
No					1.00 (reference)
Yes	0.42	0.81	0.51	0.607	1.52 (0.31–7.41)
ECOG score					
0					1.00 (reference)
1	−0.56	0.62	−0.90	0.370	0.57 (0.17–1.94)
2	0.94	0.88	1.08	0.281	2.57 (0.46–14.30)
3	−13.37	1,455.40	−0.01	0.993	0.00 (0.00–Inf)
ASA score					
I					1.00 (reference)
II	−0.80	1.14	−0.71	0.480	0.45 (0.05–4.17)
III	−0.43	1.26	−0.34	0.734	0.65 (0.06–7.64)
IV	−13.96	1,455.40	−0.01	0.992	0.00 (0.00–Inf)
Biliary drainage					
Yes					1.00 (reference)
No	−1.15	1.06	−1.09	0.276	0.32 (0.04–2.51)
Surgical approach					
Open surgery					1.00 (reference)
Laparoscopic surgery	−1.47	0.80	−1.83	0.068	0.23 (0.05–1.11)
Robotic surgery	−0.39	0.64	−0.61	0.542	0.68 (0.19–2.36)
Pancreatic texture					
Soft					1.00 (reference)
Firm	−3.51	0.94	−3.75	<0.001	0.03 (0.01–0.08)
Pancreatic duct diameter					
Small					1.00 (reference)
Large	−2.89	0.63	−0.4.45	<0.001	0.06 (0.016–1.9)
Tumor location					
Duodenum or ampulla of Vater					1.00 (reference)
Pancreas	053	0.86	0.62	0.540	1.70 (0.31–9.24)
Bile duct	0.83	0.82	1.01	0.312	2.29 (0.46–11.35)
Age	−0.03	0.02	−1.46	0.145	0.97 (0.93–1.01)
BMI	0.52	0.14	3.80	<0.001	1.68 (1.28–2.19)
Hemoglobin level	−0.00	0.01	−0.35	0.730	1.00 (0.98–1.02)
White blood cell count	0.07	0.07	1.00	0.318	1.07 (0.94–1.22)
Neutrophil-to-lymphocyte ratio	0.01	0.03	0.53	0.596	1.01 (0.96–1.07)
CA19-9	0.01	0.00	3.23	0.001	1.01 (1.01–1.01)
CA125	0.00	0.00	0.85	0.393	1.00 (1.00–1.01)
CEA	−0.04	0.07	−0.51	0.613	0.96 (0.83–1.11)
Plasma total bilirubin level	−0.00	0.00	−0.62	0.532	1.00 (1.00–1.00)
Surgery duration	−0.14	0.22	−0.64	0.524	0.87 (0.56–1.34)
Estimated blood loss	0.00	0.00	0.01	0.995	1.00 (0.99–1.00)
Intraoperative transfusion volume	0.00	0.00	1.00	0.318	1.00 (0.99–1.00)
Intraoperative red blood cell transfusion volume	0.25	0.26	0.97	0.098	1.28 (0.78–2.12)
Number of lymph nodes removed	0.01	0.06	0.09	0.931	1.00 (0.89–1.13)

*β* represents the regression coefficient, describing the direction and magnitude of the variable's impact on the risk of pancreatic fistula; S.E. represents the standard error used to assess the uncertainty of the estimated value; *Z* represents the test statistic; OR (95% CI) refers to the odds ratio and its 95% confidence interval; Inf represents infinity; BMI represents body mass index; ECOG represents the Eastern Cooperative Oncology Group; ASA represents the American Society of Anesthesiologists; CA represents carbohydrate antigen; CEA represents carcinoembryonic antigen.

### Multicollinearity assessment

**Figure 1:**
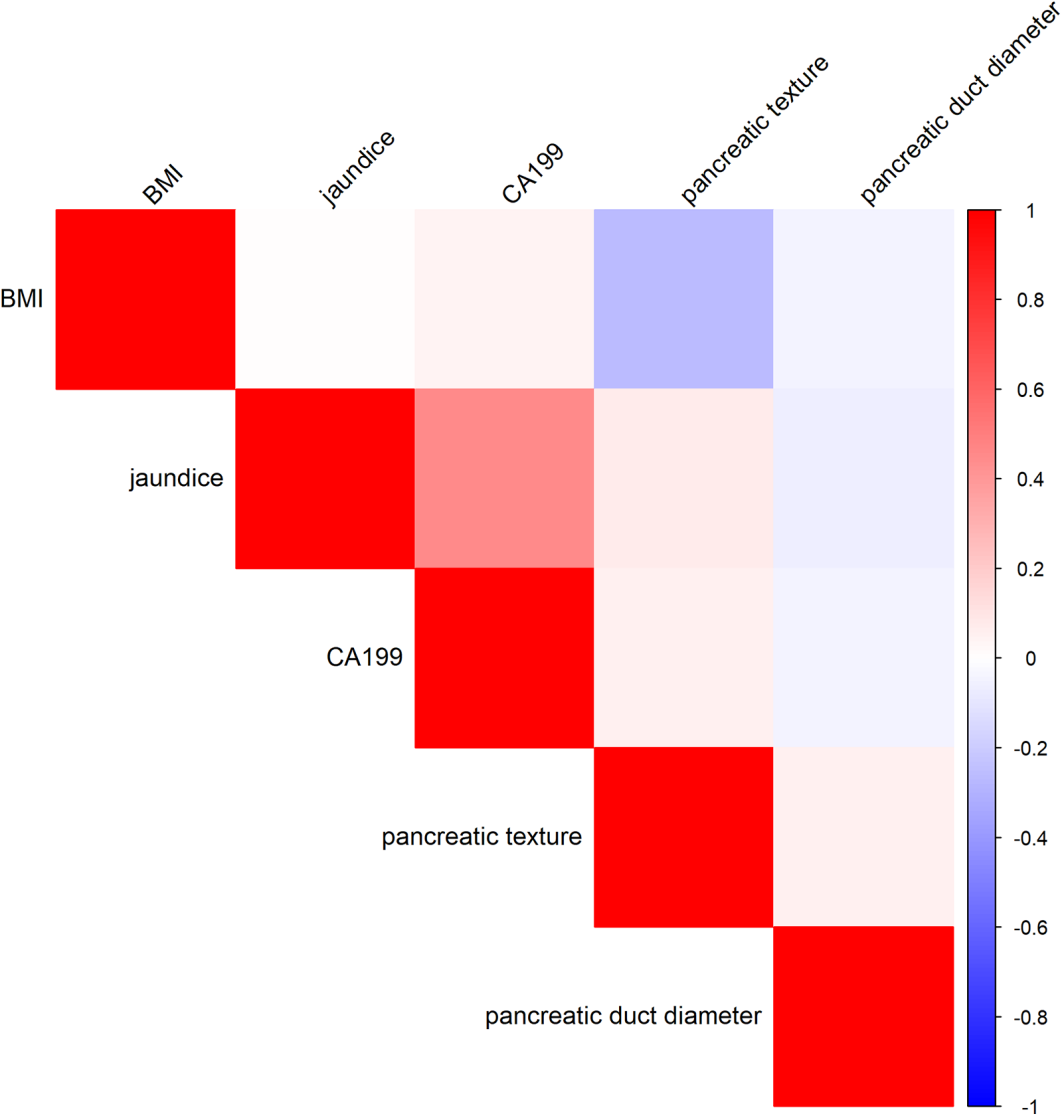
Correlation heatmap of risk factors for clinically relevant postoperative pancreatic fistula.

To evaluate potential multicollinearity among the selected independent variables (jaundice, CA19-9, pancreatic texture, and pancreatic duct diameter), the VIF was calculated using a linear regression model. The results indicated that all VIF values were below the commonly accepted threshold of 10, suggesting no severe multicollinearity in the model. The specific VIF values were as follows: Jaundice: VIF = 1.28, CA19-9: VIF = 1.27, Pancreatic Texture: VIF = 1.01, Pancreatic Duct Diameter: VIF = 1.01. It should be noted that BMI was used as the dependent variable in this linear regression model to compute the VIF values for the remaining independent variables (jaundice, CA19-9, pancreatic texture, and pancreatic duct diameter). Therefore, BMI itself does not have a corresponding VIF value in this analysis. Additionally, the correlation matrix analysis revealed that the correlation coefficients among variables were generally low, with absolute values ranging from 0.001 to 0.459. The results of the correlation matrix analysis are presented in Figure 1, The analysis of VIF and the correlation matrix indicated that the five selected variables (BMI, jaundice, CA19-9, pancreatic texture, and pancreatic duct diameter) had low levels of multicollinearity, as all VIF values were less than 1.5 and all correlation coefficients were under 0.8. This indicates that these variables demonstrated good independence in this study and can be used as independent variables in the Multivariable model for further analysis.

### Multivariable LR

**Figure 2:**
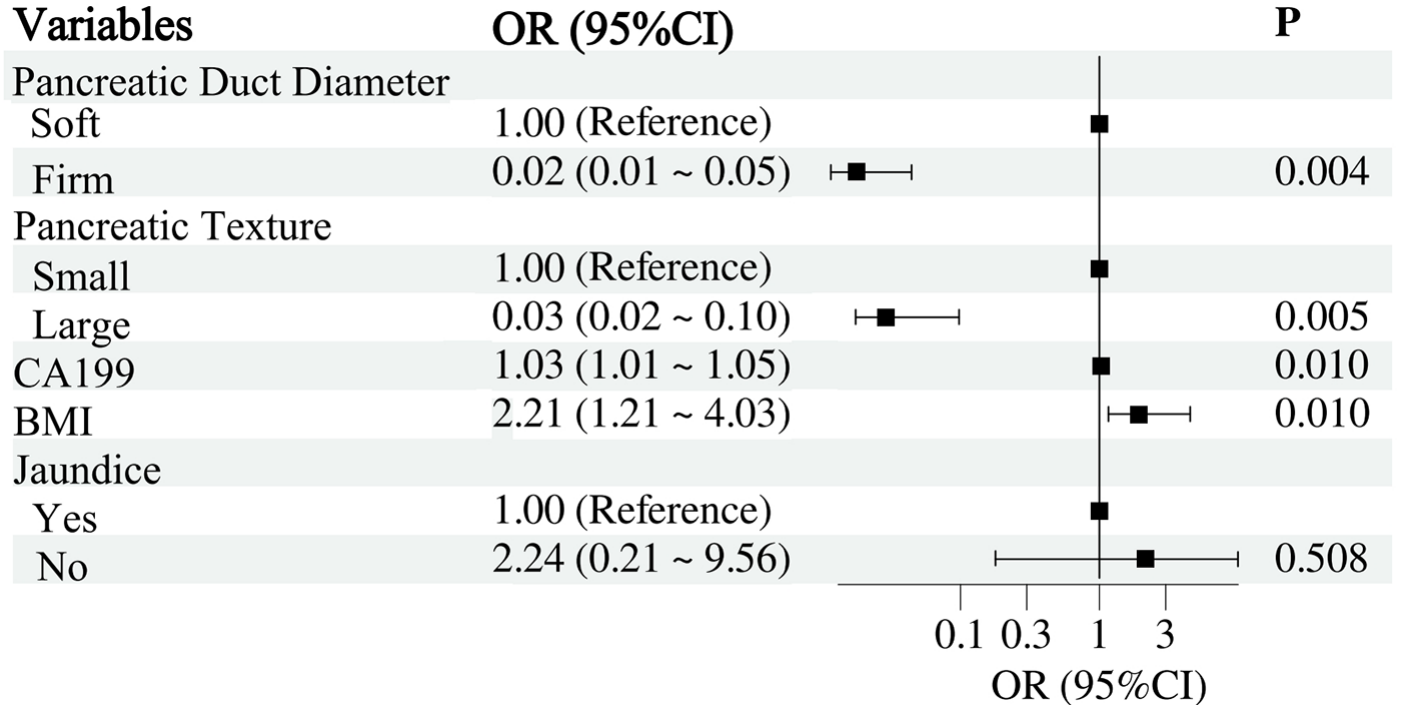
Multivariable LR analysis of factors associated with clinically relevant postoperative pancreatic fistula.

Multivariable LR analysis identified pancreatic duct diameter, pancreatic texture, CA19-9 levels, and BMI as significant independent predictors of CR-POPF (Figure 2). A smaller pancreatic duct diameter (OR = 0.02, 95% CI: 0.01–0.05, *P* = 0.004) and soft pancreatic texture (OR = 0.03, 95% CI: 0.02–0.10, *P* = 0.005) were strongly associated with an increased risk of CR-POPF. Elevated CA19-9 levels (OR = 1.03, 95% CI: 1.01–1.05, *P* = 0.010) and higher BMI (OR = 2.21, 95% CI: 1.21–4.03, *P* = 0.010) also significantly increased the risk. However, preoperative jaundice was not found to be a significant predictor (OR = 2.24, 95% CI: 0.21–9.56, *P* = 0.508). These findings suggest that pancreatic duct and texture characteristics, along with CA19-9 levels and BMI, are critical factors for predicting CR-POPF.

### RF

**Figure 3:**
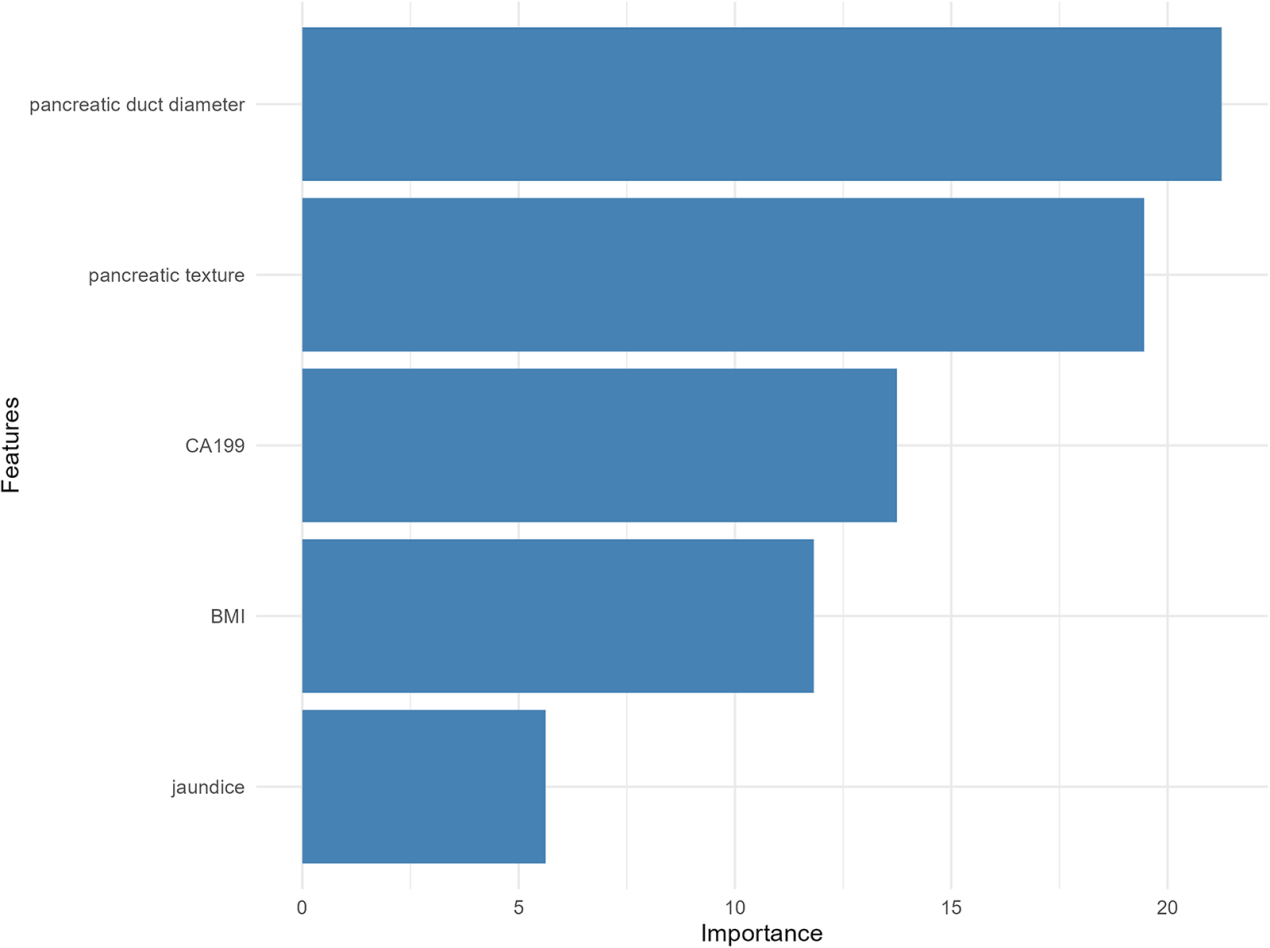
Variable importance plot from the RF model for predicting clinically relevant postoperative pancreatic fistula.

The RF model identified pancreatic duct diameter, pancreatic texture, and CA19-9 levels as the most important predictors of CR-POPF (Figure 3), with pancreatic duct diameter showing the highest importance. BMI also demonstrated moderate predictive importance, while jaundice contributed the least to the model's performance. These results underscore the critical role of pancreatic anatomy and tumor markers in accurately predicting CR-POPF risk.

### Model performance evaluation

The model performance evaluation of the multivariable LR model and the RF model is shown in Table 3. Although the RF model demonstrated reasonable discrimination and calibration, the LR model outperformed it across most metrics, suggesting a preferable balance between discrimination and calibration for CR-POPF prediction in this dataset.

**Table 3:** Model performance comparison between LR and RF.

Model	AUC (95% CI)	Accuracy % (95% CI)	Sensitivity % (95% CI)	Specificity % (95% CI)	Calibration slope ± SE	Calibration intercept ± SE
LR	0.964 (0.932–0.995)	87.4 (83.9–90.0)	48.0 (39.8–56.4)	98.0 (93.5–99.7)	1.03 ± 0.11	−0.02 ± 0.04
RF	0.901 (0.793–0.998)	81.0 (76.5–84.7)	45.5 (37.0–54.3)	92.0 (86.7–96.4)	0.95 ± 0.15	0.05 ± 0.06

Metrics are expressed as value (95% CI); calibration coefficients are estimate ± SE; bootstrap = 1,000. AUC represents the area under the curve; 95% CI refers to 95% confidence interval.

### Calibration curve

**Figure 4:**
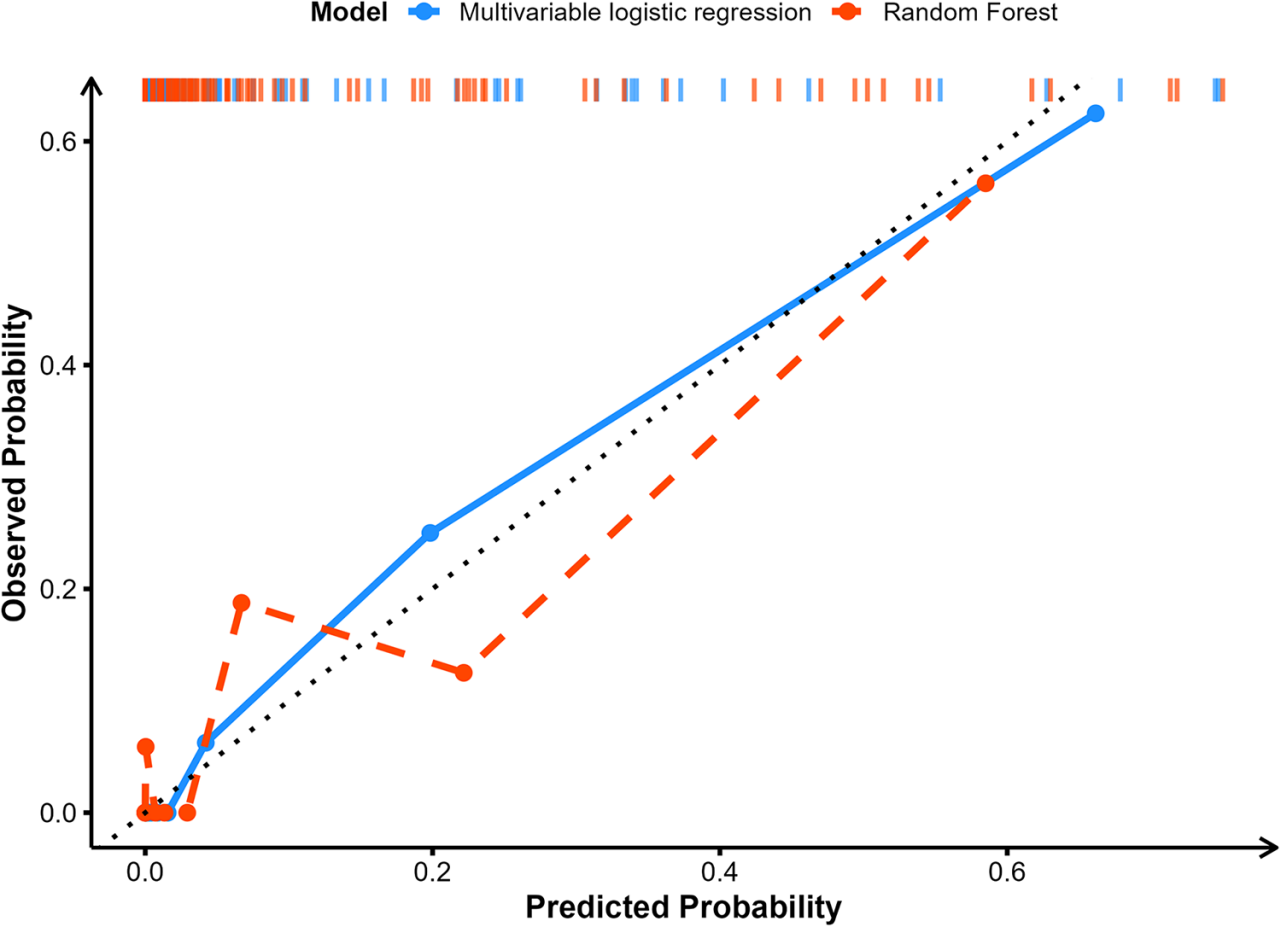
Comparison of the calibration curves for the predictive performance of the multivariable LR and RF models.

The blue solid line represents the original calibration curve of the Multivariable LR model (Figure 4). Overall, it is close to the diagonal, indicating that the predicted probabilities from multivariable LR align well with the actual incidence. There is a slight deviation in the range where the actual occurrence probability is below 0.2, but the overall trend remains stable. In the high-probability region (greater than 0.5), it closely matches the diagonal. The red solid line represents the calibration curve of the RF model. The RF shows large fluctuations in its predictions when the actual occurrence probability is below 0.2. Within the 0.2–0.5 probability band, the RF typically underestimates the actual incidence. In contrast, for probabilities above 0.5, the RF's calibration is close to the diagonal. Overall, Multivariable LR demonstrates superior calibration compared to RF, especially in the medium-to-high probability range, where its predictions align more closely with actual incidence. Meanwhile, the RF is unstable in the low-probability range.

### Receiver operating characteristic

**Figure 5:**
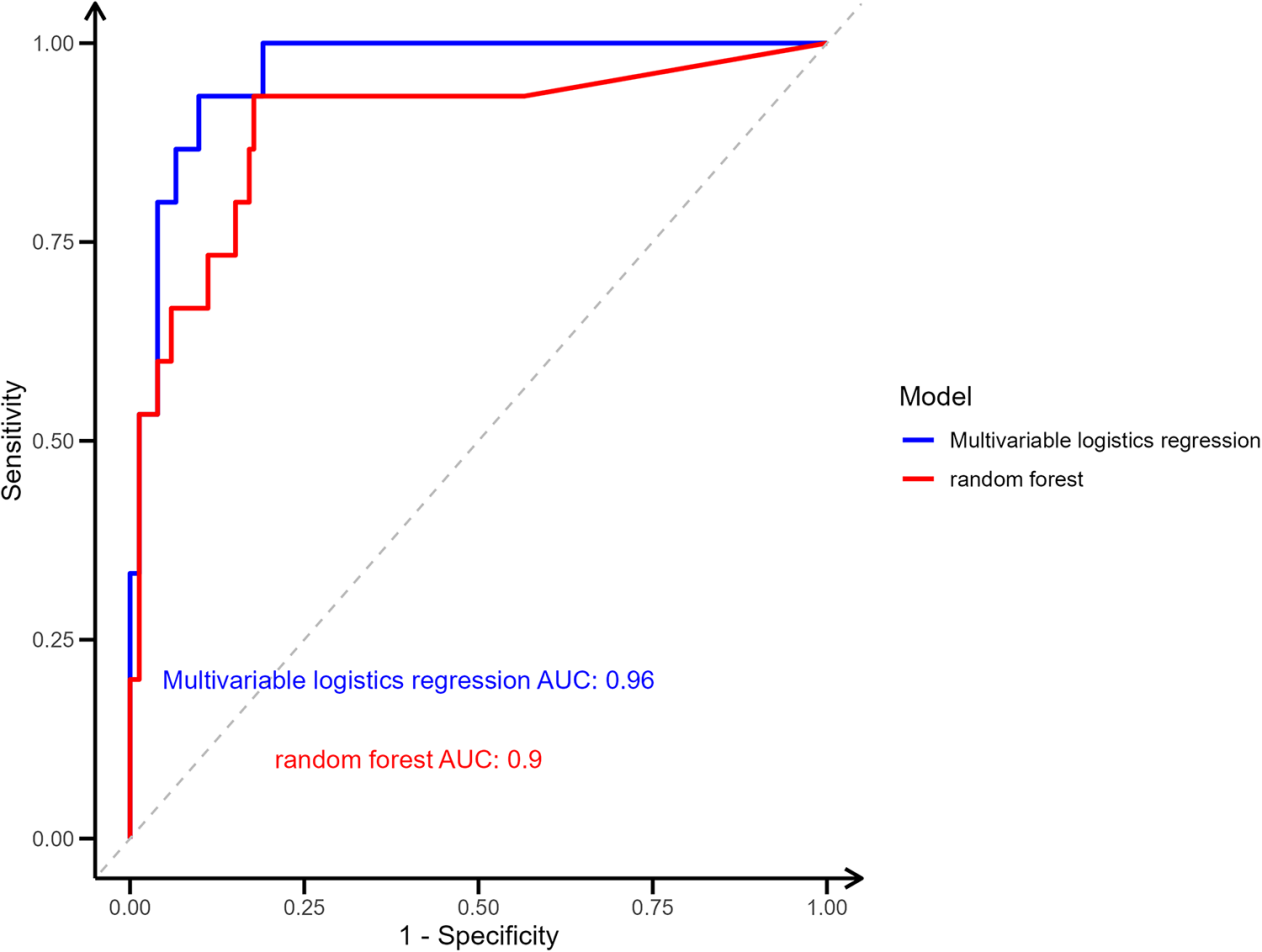
Receiver operating characteristic (ROC) curves comparing the predictive performance of LR and RF models.

The predictive performance of the Multivariable LR model and the RF model was evaluated using ROC curve analysis (Figure 5). The Multivariable LR model achieved an AUC of 0.96, Its 95% CI is 0.93–0.99, and an accuracy of 0.87 indicating excellent discriminatory power. The RF model demonstrated an AUC of 0.90, Its 95% CI is 0.79–0.99, and an accuracy of 0.81 also reflecting strong predictive ability. Comparatively, the LR model slightly outperformed the RF model in terms of AUC, suggesting its superior accuracy in predicting CR-POPF. Both models displayed robust performance, validating their utility in clinical risk prediction.

### Decision curve analysis

**Figure 6:**
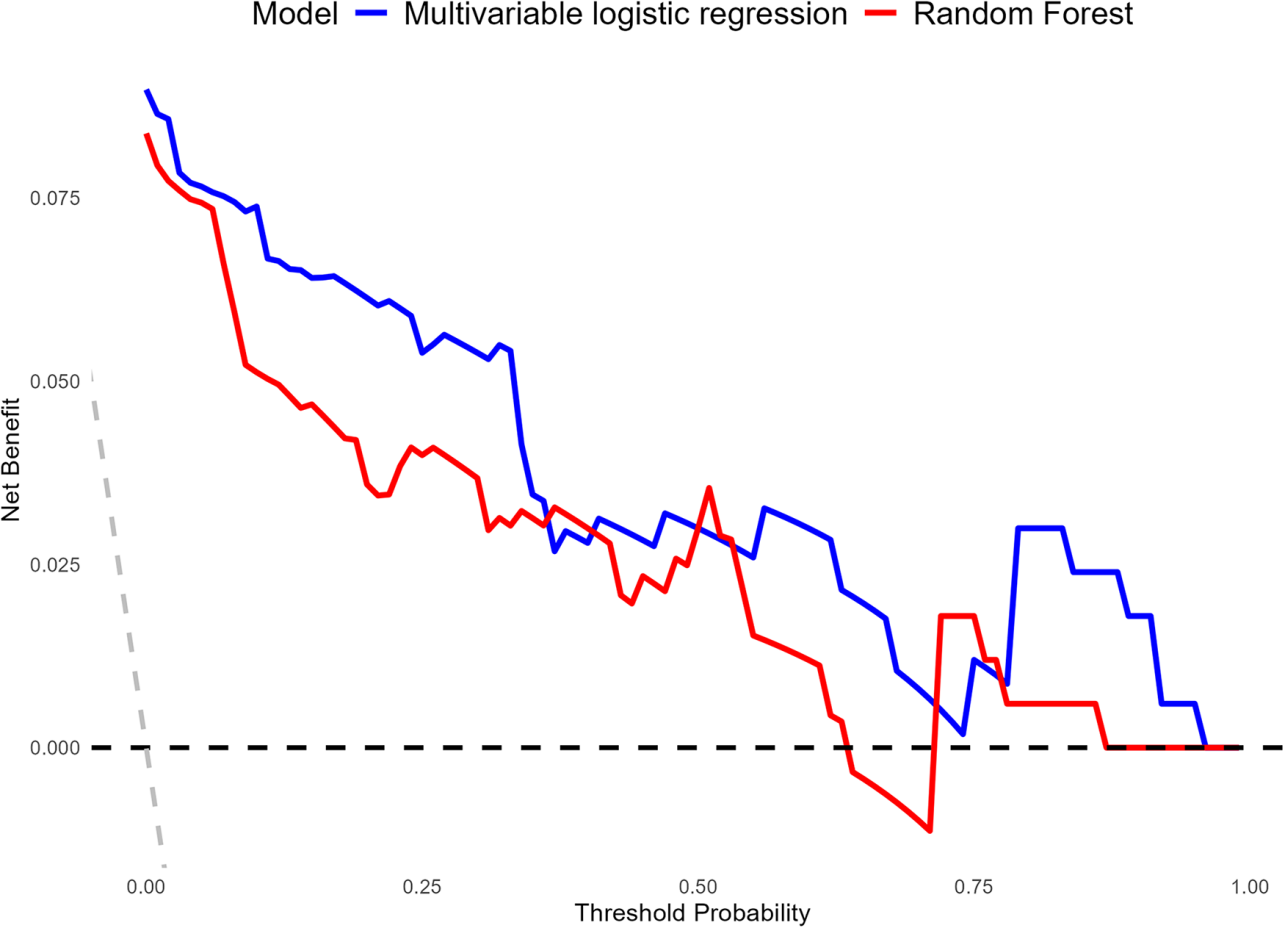
Comparison of the decision curve analysis (DCA) curves for the clinical utility of multivariable LR and RF models.

DCA was performed to evaluate the clinical utility of the Multivariable LR model and the RF model across a range of threshold probabilities (Figure 6). Both models demonstrated net benefit across clinically relevant thresholds compared to the “Treat All” and “Treat None” strategies. The Multivariable LR model consistently provided a higher net benefit compared to the RF model, particularly within the threshold probability range of 0.1–0.6. Beyond this range, both models showed comparable performance, maintaining clinical utility. These findings indicate that the Multivariable LR model offers better practical value for risk prediction and clinical decision-making in the context of CR-POPF.

## Discussion

This study identified pancreatic duct diameter, pancreatic texture, CA19-9 levels, and BMI as key independent risk factors for CR-POPF. Among these, pancreatic duct diameter and pancreatic texture were found to have the most significant influence on CR-POPF risk, consistent with prior research emphasizing the anatomical and physiological characteristics of the pancreas as critical determinants (28, 29). Elevated CA19-9 levels and higher BMI also showed a strong correlation with CR-POPF, reinforcing the role of tumor markers and patient-related factors in surgical outcomes.

The Multivariable LR model demonstrated superior predictive accuracy compared to the RF model, achieving a better calibration, a higher area under the curve (AUC) and greater clinical utility in decision curve analysis (DCA). While RF, as a machine learning algorithm, offers the advantage of handling complex variable interactions and high-dimensional datasets, its application to conventional clinical data involving a limited number of variables appears less optimal. LR, on the other hand, benefits from its simplicity, interpretability, and suitability for smaller datasets, making it more practical for clinical applications.

Several factors may explain why the LR model outperformed the RF model in our study. First, the sample size was relatively small, which may have limited the ability of the RF to capture complex interactions between variables. RF algorithms generally require large datasets to fully utilize their ability to model complex nonlinear relationships. Second, the predictor variables selected in our study primarily exhibited strong linear or near-linear associations with the outcome, as supported by both univariate and Multivariable LR analyses. The lack of complex non-linear patterns within the data may have favored the performance of LR. Third, the relatively limited number of predictor variables, coupled with low multicollinearity as confirmed by VIF analysis, likely reduced the necessity for complex ensemble methods, further enhancing the suitability of LR in this context. These data characteristics are well-aligned with the strengths of LR, explaining its superior performance in this study.

A smaller pancreatic duct diameter may lead to impaired pancreatic juice drainage, elevated intrapancreatic pressure, and an increased risk of anastomotic leakage (30). Furthermore, a smaller pancreatic duct diameter increases the complexity of the anastomosis, potentially resulting in loose sutures or incomplete duct-to-intestine anastomosis, causing pancreatic juice leakage (31). The soft pancreatic tissue is relatively loose and lacks fibrous support, making it difficult to secure during suturing (19), which compromises the stability of the anastomosis and increases the likelihood of postoperative pancreatic fluid leakage. Related research indicates that patients with soft pancreatic texture have a higher volume of pancreatic fluid secretion and increased enzyme activity (32). Clinically, pancreatic texture is influenced by underlying pathological conditions. A soft pancreatic parenchyma is commonly observed in patients with a normal pancreas or in those with periampullary tumors without significant pancreatic duct obstruction. In contrast, a firm pancreatic texture is typically associated with chronic pancreatitis or pancreatic ductal adenocarcinoma, where longstanding inflammation or tumor-induced desmoplastic reaction results in extensive fibrosis and parenchymal atrophy (33, 34). Therefore, the underlying disease processes may significantly affect the risk of postoperative CR-POPF by impacting the mechanical properties of the pancreas. If these fluids leak, they can cause enzymatic autodigestion of the surrounding tissues, resulting in pancreatic fistula. High levels of CA19-9 are usually indicative of more severe pancreatic inflammation or tumor burden, such as pancreatic cancer or biliary obstruction (35–37). These pathological conditions may weaken pancreatic tissue, making postoperative pancreatic fistula more likely. BMI is recognized as a reliable indicator of protein-calorie malnutrition and obesity. A research found that BMI is correlated with CR-POPF, possibly because a higher BMI is associated with increased visceral fat in the pancreas (38), which softens and weakens the pancreatic texture, significantly increasing the difficulty of pancreatoenteric anastomosis and the risk of CR-POPF. Elis (39) also demonstrated that BMI ≥30 kg/m^2^ is a risk factor for CR-POPF. Studies by Le Bian (40) and Zou (41) found that BMI ≥25 kg/m^2^ is a risk factor for CR-POPF. Despite the lack of statistical significance of preoperative jaundice status in Multivariable LR and RF analyses, related studies indicate a strong link between preoperative jaundice and CR-POPF. Research has shown that preoperative TB >250 μmol/L warrants biliary drainage to reduce bilirubin levels, thereby significantly decreasing the occurrence of CR-POPF. Chen et al. (42) through a multicenter retrospective analysis of 1,465 patients undergoing PD, concluded that preoperative biliary drainage decreases the risk of CR-POPF, underscoring the impact of preoperative TB levels on CR-POPF incidence. Research conducted by Xi Yiqing and Shen et al. (43, 44), similarly concluded that elevated preoperative serum bilirubin levels are a risk factor for postoperative pancreatic fistula.

Despite these limitations, this study also presents several novel contributions to the field. This study systematically compares the predictive performance of a multivariable LR model and RF model for CR-POPF in an East Asian cohort. It provides a multidimensional evaluation of model performance. Moreover, this study employs robust internal validation to enhance the reliability of the results. Finally, by focusing on routine preoperative and intraoperative variables, our study developed a clinically applicable model that can assist PD patients in real-time risk stratification and personalized perioperative management. These features contribute to the methodological rigor and practical significance of our findings. Using the LR-based risk score, patients can be stratified into low and high CR-POPF-risk categories, allowing peri-operative management to be individualized. Preoperatively, patients predicted to have a high risk of CR-POPF receive biliary drainage, metabolic optimisation. Intraoperatively, if the patient is predicted to be high risk, surgical modifications, such as reinforced suturing, can be considered. Postoperatively, for high-risk patients, extending the duration of drainage and providing anti-infective therapy can be considered.

While this study provides valuable insights into the risk factors for CR-POPF, the relatively small sample size and limited scope of analyzed variables may constrain the generalizability of the findings. External validation was not performed in this study due to the limited sample size available. Therefore the generalizability and real-world predictive performance of our models remain to be confirmed by future external validation studies. Additionally, the retrospective design inherently introduces unmeasured confounding variables, which may affect the internal validity of the findings. Further large-scale, prospective studies are warranted to validate these results. Future research should explore the integration of novel biomarkers and advanced imaging techniques for more precise preoperative risk assessment. Additionally, the application of artificial intelligence in the prediction and management of CR-POPF warrants further investigation.

## Conclusion

This study identified pancreatic duct diameter, pancreatic texture, CA19-9 levels, and BMI as key risk factors for CR-POPF. The Multivariable LR model demonstrated better predictive performance and greater clinical utility compared to the RF model, as confirmed by Calibration curve analysis, Receiver Operating Characteristic and Decision Curve Analysis. These findings highlight the importance of incorporating anatomical, biochemical, and clinical factors into risk assessments to enhance surgical outcomes.

## Data Availability

The raw data supporting the conclusions of this article will be made available by the authors, without undue reservation.

## References

[1] Wang M Peng B Liu J Yin X Tan Z Liu R Practice patterns and perioperative outcomes of laparoscopic pancreaticoduodenectomy in China: a retrospective multicenter analysis of 1029 patients. Ann Surg. (2021) 273(1):145–53. 10.1097/SLA.0000000000003190 30672792

[2] Clancy TE Ashley SW. Pancreaticoduodenectomy (whipple operation). Surg Oncol Clin N Am. (2005) 14(3):533–52, vii. 10.1016/j.soc.2005.05.006 15978428

[3] Torres O Moraes-Junior J Fernandes E Hackert T. Surgical management of postoperative grade C pancreatic fistula following pancreatoduodenectomy. Visc Med. (2022) 38(4):233–42. 10.1159/000521727 36160826 PMC9421704

[4] Vollmer CM Jr Sanchez N Gondek S McAuliffe J Kent TS Christein JD A root-cause analysis of mortality following major pancreatectomy. J Gastrointest Surg. (2012) 16(1):89–102; discussion 102–3. 10.1007/s11605-011-1753-x 22065319

[5] Casciani F Bassi C Vollmer CM. Decision points in pancreatoduodenectomy: insights from the contemporary experts on prevention, mitigation, and management of postoperative pancreatic fistula. Surgery. (2021) 170(3):889–909. 10.1016/j.surg.2021.02.064 33892952

[6] Breiman L. Random forests. Mach Learn. (2001) 45:5–32. 10.1023/A:1010933404324

[7] Enzer NA Chiles J Mason S Shirahata T Castro V Regan E Proteomics and machine learning in the prediction and explanation of low pectoralis muscle area. Sci Rep. (2024) 14(1):17981. 10.1038/s41598-024-68447-y 39097658 PMC11297919

[8] Hornung R Wright MN. Block forests: random forests for blocks of clinical and omics covariate data. BMC Bioinformatics. (2019) 20(1):358. 10.1186/s12859-019-2942-y 31248362 PMC6598279

[9] Shehab M Abualigah L Shambour Q Abu-Hashem MA Shambour MK Alsalibi AI Machine learning in medical applications: a review of state-of-the-art methods. Comput Biol Med. (2022) 145:105458. 10.1016/j.compbiomed.2022.105458 35364311

[10] Ahsan MM Luna SA Siddique Z. Machine-learning-based disease diagnosis: a comprehensive review. Healthcare. (2022) 10(3):541. 10.3390/healthcare10030541 35327018 PMC8950225

[11] Mungroop TH van Rijssen LB van Klaveren D Smits FJ Van Woerden V Linnemann RJ Alternative fistula risk score for pancreatoduodenectomy (a-FRS): design and international external validation. Ann Surg. (2019) 269(5):937–43. 10.1097/SLA.0000000000002620 29240007

[12] Li Y Zhou F Zhu DM Yang J Yao J Wei YJ Novel risk scoring system for prediction of pancreatic fistula after pancreaticoduodenectomy. World J Gastroenterol. (2019) 25(21):2650–64. 10.3748/wjg.v25.i21.2650 31210716 PMC6558436

[13] He C Zhang Y Li L Zhao M Wang C Tang Y. Risk factor analysis and prediction of postoperative clinically relevant pancreatic fistula after distal pancreatectomy. BMC Surg. (2023) 23:5. 10.1186/s12893-023-01907-w 36631791 PMC9835372

[14] Li B Pu N Chen Q Mei Y Wang D Jin D Comprehensive diagnostic nomogram for predicting clinically relevant postoperative pancreatic fistula after pancreatoduodenectomy. Front Oncol. (2021) 11:717087. 10.3389/fonc.2021.717087 34277458 PMC8281206

[15] Ouyang L Liu RD Ren YW Nie G He TL Li G Nomogram predicts CR-POPF in open central pancreatectomy patients with benign or low-grade malignant pancreatic neoplasms. Front Oncol. (2022) 12:1030080. 10.3389/fonc.2022.1030080 36591477 PMC9797993

[16] Wang GQ Yadav DK Jiang W Hua YF Lu C. Risk factors for clinically relevant postoperative pancreatic fistula (CR-POPF) after distal pancreatectomy: a single center retrospective study. Can J Gastroenterol Hepatol. (2021) 2021:8874504. 10.1155/2021/8874504 33542910 PMC7840268

[17] Yang F Windsor JA Fu DL. Optimizing prediction models for pancreatic fistula after pancreatectomy: current status and future perspectives. World J Gastroenterol. (2024) 30:1329–45. 10.3748/wjg.v30.i10.1329 38596504 PMC11000089

[18] Ashraf Ganjouei A Romero-Hernandez F Wang JJ Casey M Frye W Hoffman D A machine learning approach to predict postoperative pancreatic fistula after pancreaticoduodenectomy using only preoperatively known data. Ann Surg Oncol. (2023) 30:7738–47. 10.1245/s10434-023-14041-x 37550449

[19] Bassi C Marchegiani G Dervenis C Sarr M Hilal MA Adham M The 2016 update of the international study group (ISGPS) definition and grading of postoperative pancreatic fistula: 11 years after. Surgery. (2017) 161(3):584. 10.1016/j.surg.2016.11.014 28040257

[20] Shen J Guo F Sun Y Zhao J Hu J Ke Z Predictive nomogram for postoperative pancreatic fistula following pancreaticoduodenectomy: a retrospective study. BMC Cancer. (2021) 21(1):550. 10.1186/s12885-021-08201-z 33992090 PMC8126152

[21] James G Witten D Hastie T Tibshirani R. An Introduction to Statistical Learning. New York: Springer (2013).

[22] Franzese M Iuliano A. Correlation analysis. In: Ranganathan S Gribskov M Nakai K, editors. Encyclopedia of Bioinformatics and Computational Biology. Amsterdam: Elsevier (2019). p. 706–21.

[23] Glonek GFV McCullagh P. Multivariate logistic models. J R Stat Soc Series B Stat Methodol. (1995) 57(3):533. 10.1111/j.2517-6161.1995.tb02046.x

[24] Rigatti SJ. Random forest. J Insur Med. (2017) 47(1):31. 10.17849/insm-47-01-31-39.1 28836909

[25] Huang Y Li W Macheret F Gabriel RA Ohno-Machado L. A tutorial on calibration measurements and calibration models for clinical prediction models. J Am Med Inform Assoc. (2020) 27(4):621–33. 10.1093/jamia/ocz228 32106284 PMC7075534

[26] Eng J. Receiver operating characteristic analysis: a primer1. Acad Radiol. (2005) 12(7):909. 10.1016/j.acra.2005.04.005 16039544

[27] Fitzgerald M Saville BR Lewis RJ. Decision curve analysis. J Am Med Assoc. (2015) 313(4):409. 10.1001/jama.2015.37 25626037

[28] Zhang B Yuan Q Li S Xu Z Chen X Li L Risk factors of clinically relevant postoperative pancreatic fistula after pancreaticoduodenectomy: a systematic review and meta-analysis. Medicine. (2022) 101(26):e29757. 10.1097/MD.0000000000029757 35776984 PMC9239615

[29] Schuh F Mihaljevic AL Probst P Trudeau MT Müller PC Marchegiani G A simple classification of pancreatic duct size and texture predicts postoperative pancreatic fistula: a classification of the international study group of pancreatic surgery. Ann Surg. (2023) 277(3):e597–608. 10.1097/SLA.0000000000004855 33914473 PMC9891297

[30] Yang JX Ye SY Dai D. Risk factors and preventive measures for postoperative pancreatic fistula after pancreaticoduodenectomy. World Chin J Dig. (2020) 28:914–9. 10.11569/wcjd.v28.i18.914

[31] Barreto SG Shukla PJ. Different types of pancreatico-enteric anastomosis. Transl Gastroenterol Hepatol. (2017) 2:89. 10.21037/tgh.2017.11.02 29264427 PMC5723729

[32] Wellner UF Kayser G Lapshyn H Sick O Makowiec F Höppner J A simple scoring system based on clinical factors related to pancreatic texture predicts postoperative pancreatic fistula preoperatively. HPB. (2010) 12(10):696. 10.1111/j.1477-2574.2010.00239.x 21083795 PMC3003480

[33] Nikolaidis P Hammond NA Day K Yaghmai V Wood CG Mosbach DS Imaging features of benign and malignant ampullary and periampullary lesions. Radiographics. (2014) 34:624–41. 10.1148/rg.343125191 24819785

[34] Kalayarasan R Himaja M Ramesh A Kokila K. Radiological parameters to predict pancreatic texture: current evidence and future perspectives. World J Radiol. (2023) 15:170–81. 10.4329/wjr.v15.i6.170 37424737 PMC10324497

[35] Bhandare MS Varty GP Reddy Obili RC Chopde A Pawar A Krishnakumar K CA 19-9 surveillance detects recurrences early and contributes to improvement in survival in resected ampullary cancers: analysis of 572 cases. Ann Surg. (2024). 10.1097/SLA.0000000000006419 38939924

[36] Yasui K Yoshida R Umeda Y Kuise T Yoshida K Takagi K Sustained elevation of CA19-9 after resection is a strong prognostic factor for resectable pancreatic cancer. HPB. (2021) 23:S254. 10.1016/j.hpb.2020.11.640

[37] Izumo W Higuchi R Furukawa T Yazawa T Uemura S Shiihara M Evaluation of preoperative prognostic factors in patients with resectable pancreatic ductal adenocarcinoma. Scand J Gastroenterol. (2019) 54(6):780. 10.1080/00365521.2019.1624816 31180790

[38] Nishida Y Kato Y Kudo M Aizawa H Okubo S Takahashi D Preoperative sarcopenia strongly influences the risk of postoperative pancreatic fistula formation after pancreaticoduodenectomy. J Gastrointest Surg. (2016) 20(9):1586. 10.1007/s11605-016-3146-7 27126054

[39] Ellis RJ Brock Hewitt D Liu JB Cohen ME Merkow RP Bentrem DJ Preoperative risk evaluation for pancreatic fistula after pancreaticoduodenectomy. J Surg Oncol. (2019) 119(8):1128. 10.1002/jso.25464 30951614 PMC6894415

[40] Zarzavadjian Le Bian A Fuks D Montali F Cesaretti M Costi R Wind P Predicting the severity of pancreatic fistula after pancreaticoduodenectomy: overweight and blood loss as independent risk factors: retrospective analysis of 277 patients. Surg Infect. (2019) 20(6):486. 10.1089/sur.2019.027 31063046

[41] Zou SY Wang WS Zhan Q Deng XX Shen BY. Higher body mass index deteriorates postoperative outcomes of pancreaticoduodenectomy. Hepatobiliary Pancreat Dis Int. (2020) 19(2):163. 10.1016/j.hbpd.2019.11.007 31862346

[42] Chen H Wang W Ying X Deng X Peng C Cheng D Predictive factors for postoperative pancreatitis after pancreaticoduodenectomy: a single-center retrospective analysis of 1465 patients. Pancreatology. (2020) 20(2):211. 10.1016/j.pan.2019.11.014 31831390

[43] Xi YQ Wei X Yang ZS Wang HQ Wang X Yang TC A meta-analysis of the risk factor of pancreatic fistula after pancreaticoduodenectomy. Chin J Exp Surg. (2019) 36:1857–60. 10.3760/cma.j.issn.1001-9030.2019.10.037

[44] Shen Z Zhang J Zhao S Zhou Y Wang W Shen B. Preoperative biliary drainage of severely obstructive jaundiced patients decreases overall postoperative complications after pancreaticoduodenectomy: a retrospective and propensity score-matched analysis. Pancreatology. (2020) 20(3):529. 10.1016/j.pan.2020.02.002 32107192

